# Hospitalisation expenditure on tuberculosis among tribal populations in India: A repeated cross-sectional analysis of national sample survey data, 2004 to 2018

**DOI:** 10.1016/j.puhip.2024.100490

**Published:** 2024-03-05

**Authors:** Denny John, Jeetendra Yadav, Devdatta Ray, Paramita Bhattacharya, Nirmalya Mukherjee, Rajan Patil, Vivek Varma, Sahadeb Hembram, Moumita Hansda

**Affiliations:** a Faculty of Life and Allied Health Sciences, MS Ramaiah University of Applied Sciences, Bengaluru, India; b ICMR-National Institute of Medical Statistics, New Delhi, India; c Manipal Academy of Higher Education, Manipal, India; d Centre for Public Health Research, MANT, Kolkata, India; e KLE University, Belgaum, India; f Assam University, Silchar, India; g MANT, Kolkata, India

## Abstract

**Objective:**

Tribal population in India (8.6% of the total population) have a greater prevalence of tuberculosis compared to the national average. The article aims to study out-of-pocket expenditure (OOPE), hardship financing, and impoverishment effects of TB hospitalisation treatment among tribal populations in India.

**Methods:**

Data of three rounds of National Sample Surveys (NSS) 60th (2004–05), 71st (2013–14) and 75th (2017–18) rounds were analyzed. Descriptive statistics, bivariate estimates and multivariate models were performed to calculate the OOPE, healthcare burden (HCB), catastrophic health expenditure (CHE), hardship financing and impoverishment effects using standard definitions at February 2023 price values. Propensity score matching (PSM) was used to examine the effect of health insurance coverage on catastrophic health expenditure, and impoverishment.

**Results:**

Over two-thirds of the TB cases are seen in the economically productive age group (14–59 years). Substantial OOPE and its impact on HCB, CHE, and poverty impact observed among 15–35 age group across all three rounds. Illiterate patients and those availing private hospitals for TB treatment had higher OOPE, HCB, hardship financing, CHE, and poverty impact. 38.5% (2014) and 33.2% (2018) are covered with any kind of public healthcare coverage, PSM analysis shows households with health insurance have lower incidence of CHE and impoverishment effects due to TB hospitalisation expenditure.

**Conclusions:**

The current study aids in comprehending the patterns in the financial burden of TB on tribal households during the previous 15 years and gives policy makers information for efficient resource allocation management for TB among Indian tribal communities.

## 1 Introduction

*Mycobacterium tuberculosis* infection causes the highest number of deaths due to infectious diseases in the world [[1], [2], [3]]. India has always been at the forefront of TB control, yet it still has the highest burden of TB cases (27% of the global share) in the world [4]. India also has the highest burden of multi-drug-resistant tuberculosis (MDR-TB) [[5], [6], [7]]. Additionally, MDR-TB calls for a higher cost of treatment than drug-resistant TB, this adds to the financial burden for TB management [8].

In India, tribal population (110.4 million people comprising of 8.6% of total population) have higher prevalence (703 per 100,000) of TB compared to national average (256 per 100,000) [9]. There also exists a wide variation of TB prevalence among the tribal groups at regional and state levels [10]. 10.4% of all TB notified patients in the country belong to tribal communities [9,11,12].

Since 2005 the National TB program have prioritized tribal subgroup population through Tribal Action Plans [13]. This Tribal TB initiative is a unique partnership between the Ministry of Health and Family Welfare and Ministry of Tribal Affairs to improve TB care and support services amongst the tribal populations [13]. Despite these efforts, there are still a number of barriers to the prevention and treatment of tuberculosis in tribal populations in the country. These include poverty, malnutrition, illiteracy, alcoholism, substance abuse, poor access to health care, and a variety of socio-cultural and environmental factors that affect the access and duration of TB treatments [[11], [12], [13]] There also exist other factors such as rituals, beliefs about herbal medicines, lack of community involvement, barriers in the health system (such a lack of personnel with the right training), and communication and cultural divides between the patient and the caregiver [[11], [12], [13]]. The implementation of the Revised National Tuberculosis Control Program (RNTCP) has been shown to be hindered by social stigma and a lack of awareness of TB among tribal people [10,14].

Previous studies have highlighted TB to be a major cause of catastrophic health expenditure for the tribal populace in the country. Muniyandi et al. (2020) highlighted the need for the decrease of direct and indirect TB patient costs so as to prevent chances of catastrophic payments to particularly vulnerable tribal groups (PVTGs) [15]. Although majority of the PVTGs received treatment free of costs, those who incurred costs had to face catastrophic TB care expenditure amounting to 10% of various costs in relation to annual family income [15].

Studies focusing on healthcare expenditure on TB in India previously have looked at tribal populations however these are limited to a specific geographical area [10,15,16]. Other studies that have used nationally representative surveys such as the National Sample Survey (NSS) have included tribal populations but have not described the various socio-economic dimensions, such as age, education, gender, marital status, income quintile etc. [[17], [18], [19]]. In India, tribal populations have higher prevalence with over 10% of all TB notified patients belonging to tribal communities [10,14,15]. The financial impact of hospitalisation related to out-of-pocket expenses (OOPE) caused by tuberculosis (TB) among tribal populations can be influenced by several factors; including literacy, religion, caste, urban/rural residence, type of ward (paying, general, paying special, and free), and length of hospital stay.

Our study is specifically focused on tribal populations in India, across time periods using a repeated cross-sectional analysis of the 60th, 71st and 75th rounds of NSS across socio-economic and demographic characteristics, for TB-related hospitalisation out-of-pocket expenditure (OOPE), catastrophic health expenditure, hardship financing, and impoverishment effects. There is need to highlight the financial impact of hospitalisation and its various socio-economic dimensions related OOPE due to TB among tribal populations for bringing forth attention to this underserved population for the policy makers in the country.

## 2 Methods

### 2.1 Data and sample size

This study used secondary data from three rounds of NSS (60th round (2004), 71st round (2014) and 75th round (2018)). NSS 60th round data covered 73,868 households and 383,338 individuals, NSS 71st round covered 65,932 households and 333,104 individuals and NSS 75th round covered 1,13,823 households and 5,55,115 individuals. For our analysis, all the tribal population who were hospitalized due to TB (114 in NSS 60th round, 106 in NSS 71st round and 115 in NSS 75th round) in the last one year preceding the survey were included.

### 2.2 Study design

NSS is a cross-sectional, nationally representative, large-scale survey in India which adopts a multi-stage sampling design to select a representative sample of households across all states and union territories in India. In the first stage of the sampling, villages/urban blocks are selected using probability proportional to size with replacement. In the second stage, households are chosen by systematic random sampling without replacement (from selected villages/urban blocks). More ssampling design, data collection, and management procedures are described in the respective NSS reports [[20], [21], [22]].

### 2.3 Outcome measurements

For OOPE, this study used direct medical costs (hospital stay, consultation, treatment medicines and procedures, laboratory and other investigation charges), direct non-medical costs (transportation, meals, lodging (for patients and attendants) [[16], [17], [18]]. NSS captures hospitalisation expenditure for the last 365 days preceding the survey period. Respective sample weights were then applied on hospitalisation expenditure for TB for the calculation of results [[16], [17], [18]].

The cost of treatment on TB as a share of total HCE of households and reported as Healthcare Burden was calculated using the formula below [17,18];

$$\text{Share}\;\text{of}\;\text{OOPE}\;\text{on}\;\text{HCE}=\frac{\;\text{Household}’s\;\text{Yearly}\;\text{Health}\;\text{Care}\;\text{Expenditure}\;x\;100}{\text{Household}’s\;\text{Yearly}\;\text{Consumption}\;\text{Expenditure}}$$

Our study estimates the ratio method for CHE that if OOPE exceeding 10% of total HCE as suggested in previous studies [[17], [18], [19]].

$$CHE=1\;if\;OOPE>{10\%\;\text{HCE}}_{i}\;and\;CHE=0\;if\;OOPE\leq {10\%\;\text{HCE}}_{i}\text{,}$$

Hardship financing is a situation when a household have to borrow money with interest or sell its property/assets to meet its health care expenses [23].

The impoverishment effects of OOPE due to TB was measured using two indices: poverty head count ratio and poverty gap ratio. The poverty gap measures the percentage deficit from the poverty line of those households that have become poor due to OOPE, and poverty gap ratio measures the percentage deficit from the poverty line of households that have become poor due to OOPE as a proportion of all the households in the population [[17], [18], [19]].

When HCE is equal or more than poverty line (PL) and after making the OOPE (HCE-OOPE) is less than the poverty line (PL) is called a poverty head count (PHC) [[17], [18], [19]]. For the poverty line, this study used, per capita monthly expenditure’ as ₹972 in rural and ₹1407 in urban according the Rangarajan Committee, 2014 [24]. The amount of poverty line is adjusted for inflation to February 2023 using the consumer price index as the Rangarajan Committee recommendation is based on 2012 data.

$$Poverty\;head\;count\;\left({PHC}_{i}\right)=1\;if\;{HCE}_{i}\geq {PL}_{i}\&\left({HCE}_{i}-{OOPE}_{i}\right)<{PL}_{i}$$

$$Otherwise\;0,where\;{HCE}_{i}\;is\;monthly\;consumption\;expenditure\;of\;i^{th}\;household\text{.}$$

Percentage of households who falls below poverty line due to OOPE is defined as poverty head count ratio (PHCR) [[17], [18], [19]].

$$Poverty\;head\;count\;ratio\left(PHCR\right)=\frac{\sum_{i=1}^{N}{\text{HH}\;\text{Impoverishment}}_{i}}{\text{Total}\;\text{households}\;\left(N\right)}\times 100$$

The poverty gap ratio (PGR) is measured the average percentage deﬁcit from the poverty line due to OOPE [18,19].

$$Povery\;gap\;\left({PG}_{i}\right)={\text{HH}\;\text{Impoershument}}_{i}\times \left\{{PL}_{i}-\;\left({HCE}_{i}-{OOPE}_{i}\right)\right\}/{PL}_{i}$$

$$Povery\;gap\;ratio\;\left(\;PGR\right)=\frac{1}{Total\;HH\;\left(N\right)}\sum_{i=1}^{N}{Poverty\;gap}_{i}\times 100$$

Currency exchange rate of 1 USD=Rupee 82.6321 (Average exchange rate in February 2023) was considered for conversion for presentation of results.

### 2.4 Defining predictor variables

Important socioeconomic and demographic predictors such as sex, age, education, caste, religion, income quintile, place of residence, regions of residence, types of health facility used for treatment, types of wards used for treatment and duration of hospitalisation were included as predictor variables in the present study based on the determinants examined in earlier similar studies and available variables in the dataset [17,19].

### 2.5 Analytical approach

Descriptive statistics, bivariate estimates and multivariable models were performed in order to achieve the research objectives. In the first part of the analysis, descriptive analysis was carried out to estimate the mean OOPE and mean yearly consumption expenditure. In the second step of the analysis, bivariate analyses were carried out to understand the differences in health care burden, catastrophic health expenditure, and hardship financing by socioeconomic characteristics of the patient. multivariable linear regression for OOPE (as a continuous variable) and logistic regression analysis for CHE and hardship financing (as a binary variable) were carried out to estimate the adjusted effects of selected covariates for hospitalisation only which used by many previous studies [17,19]; as sample sizes in population utilizing OP care was limited. To take into account the survey design, i.e., sampling weights with clustering and strata while estimating bivariate and multivariable statistics, the SVY command [25] was used in STATA 13 (StataCorp LLP) [26]. In order to compare the expenditure of three rounds Consumer Price Index (CPI) has been used, usually termed as “*Inflation Rate*”, which measures and examines the weighted average of prices of a basket of consumer goods and services, such as transportation, food, and medical care. All the expenditure reported in the year 2004 and 2014 and 2017-18 were further adjusted for inflation to February 2023 using the consumer price index with the following formula [18,19].

Cost in February 2023=(Consumer price index for Cost in February 2023)/(Average consumer price index of March and April 2004)*Amount of treatment
Cost in Cost in February 2023=(Consumer price index for Cost in February 2023)/(Average consumer price index of March and April 2014)*Amount of treatment
Cost in February 2023=(Consumer price index for Cost in February 2023)/(Average consumer price index of December 2017 and January 2018)*Amount of treatment

Propensity score matching (PSM) was used to examine the effect of health insurance coverage on catastrophic health expenditure, and impoverishment [27]. In our study, PSM establishes that an intervention (households covered with any health insurance) contributes to an outcome of interest (impact of OOPE that is the incidence of CHE), i.e., ‘does household covered by any health insurance has lower incidence of CHE and impoverishment?’. The average treatment on treated (ATT), which measures the average difference in CHE and poverty impact that health insurance schemes afford to households with health care coverage, is a measure of the effectiveness of the health care insurance [27]. As NSS 60th round did not report information on health insurance coverage, our analysis focused on NSS 71st round and NSS 75th round.

## 3 Results

Age cohort 36–59 years had the highest number of hospitalized TB patients (48.2% and 37.1% respectively) in NSS 60th round (2004) and NSS 75th round (2018) respectively, compared to age cohort 15–35 years in NSS 71st round (2014) [Table 1 and S1]. Illiterate tribals and poorest quintile reported higher percentage with hospitalisation due to TB as per NSS 60th round (2004) and NSS 71st round (2014). Rural tribals reported higher hospitalisation cases due to TB than their urban counterparts between 2004 and 2018.

**Table 1:** Profile of tuberculosis-affected tribal individuals by their selected socioeconomic and demographic characteristics in India, (NSS 2004–2018).

Background Characteristics	NSS-2004 (n = 114)		NSS-2014 (n = 106)		NSS-2018(n = 115)	
	%	n	%	n	%	n
**Age (in years)**						
0–14	4.1	7	6.9	11	8.1	10
15–35	26.6	38	45.2	45	35.6	34
36–59	48.2	48	26.8	25	37.1	53
60 and above	21.1	21	21.1	15	19.2	18
**Education**						
Illiterate	76.4	72	37.1	40	30.7	37
Up toPrimary	15.9	23	38.8	30	40.7	36
Middle	5.4	14	20.2	19	15.2	22
Secondary and above	2.2	5	3.9	7	13.4	20
**Gender**						
Male	65.2	77	60.6	56	70.3	65
Female	34.8	37	39.5	50	29.7	50
**MPCE quintile**						
Poorest	45.3	42	40.7	33	23.0	24
Poorer	13.7	17	29.5	20	23.0	22
Middle	15.2	20	13.1	19	17.2	21
Richer	18.5	22	8.2	16	27.9	22
Richest	7.2	13	8.6	18	9.1	26
**Place of residence**						
Rural	92.9	103	93.1	92	92.4	97
Urban	7.1	11	6.9	14	7.6	18
**Type of health facility**						
Public hospital	79.0	89	81.4	82	70.4	89
Private hospital	21.0	25	18.7	24	29.7	21
**Type of ward**						
Free	86.6	92	76.9	78	62.1	80
Paid	13.4	22	23.1	28	37.9	35
**Duration of stay in hospital**						
Up to 5 days	56.0	37	32.6	33	40.0	46
6–10 days	19.2	26	26.5	26	37.5	39
11 and above	24.8	51	40.9	47	22.5	30
**Health-care coverage**						
No coverage	–	–	61.5	59	66.8	77
Public	–	–	38.5	37	33.2	37
Private	–	–	–	–	0.4	1

The highest expenditure for hospitalisation was seen in the 15–35 years age group where the OOPE rose from 122.13 USD in NSS 60th round (2004) to 311.69 USD in NSS 71st round (2014), falling again to 172.38 USD in NSS 75th round (2018) (Table 2). Overall, OOPE increased from 81.81 USD in NSS 60th round (2004) to 159.97 USD in NSS 75th round (2018). Across all the three rounds, there was higher OOPE among tribal women (compared to their male counterparts), and those utilizing private health facilities. Hospitalisation-related OOPE showed more than a three-fold increase between 2004 and 2014 (i.e., from 81.81 USD to 234.41 USD) and then decreased to 159.97 USD in 2018. HCB was more prominent in rural areas than in the urban areas across all three rounds. HCB increased by 0.37% between 2004 and 2014 and then decreased by 1.76% between 2014 and 2018 (Table 2). HCB was considerably higher among those with no health insurance in 2014 but only marginally higher in 2018. Place of residence had a significant inverse association with OOPE (coef = - 0.376, 95% CI 0.084–0.082, p-value = 0.084) along with healthcare coverage (coeff = −0.328, 95% CI -0.685-0.029, p-value = 0.071) (Table 3). Type of hospital ward (i.e., paid, coef. = 0.936, 95% CI 0.494–1.378, p value = 0.00) and the stay duration (6–10 days, coef = 0.798, 95% CI 0.468–1.128, p-value = 0.00), and More than 11 days in a hospital (coef = 1.166, 95% CI 0.869–1.464, p-value = 0.00) were significant predictors for OOPE. Those with healthcare coverage were associated with significantly lower OOPE (coeff = −0.328, 95% CI -0.685-0.029, p-value = 0.071).

**Table 2:** Average Expenditure (in USD) and Health Care Burden of Tuberculosis by selected socioeconomic and demographic characteristics of the tribal patients in India (NSS 2004–2018).

Background Characteristics	NSS-2004			NSS-2014			NSS-2018		
	OOPE (in USD)	YCE (in USD)	Health Care Burden (%)	OOPE (in USD)	YCE (in USD)	Health Care Burden (%)	OOPE (in USD)	YCE (in USD)	Health Care Burden (%)
**Age (in years)**									
• 0-14	95.13	1399.03	6.8	77.66	1503.54	5.2	137.84	1513.00	9.1
• 15-35	122.13	1211.03	10.1	311.69	1384.22	22.5	172.38	1733.14	9.9
• 36-59	65.31	1219.10	5.4	251.10	1261.71	19.9	131.70	1181.85	11.1
• 60 and above	66.10	919.64	7.2	52.30	833.42	6.3	200.91	1629.85	12.3
**Education**									
Illiterate	79.45	1093.13	7.3	61.72	968.53	6.4	197.36	1182.49	16.7
Up toPrimary	88.16	1443.52	6.1	333.18	1238.50	26.9	128.44	1214.78	10.6
Middle	59.04	1346.22	4.4	315.56	1331.38	23.7	145.98	2710.93	5.4
Secondary and above	172.91	1015.82	17.0	225.83	3437.41	6.6	185.86	1650.30	11.3
**Gender**									
Male	75.68	1068.54	7.1	193.96	1113.08	17.4	177.00	1411.72	12.5
Female	93.27	1334.23	7.0	296.48	1430.91	20.7	119.70	1678.48	7.1
**MPCE quintile**									
Poorest	69.89	900.99	7.8	335.28	830.51	40.4	195.72	867.18	22.6
Poorer	143.55	1244.41	11.5	81.98	1002.35	8.2	85.28	1346.53	6.3
Middle	64.77	1427.06	4.5	208.94	1165.99	17.9	233.29	1233.08	18.9
Richer	79.57	1498.22	5.3	441.78	2221.00	19.9	168.29	1653.86	10.2
Richest	80.80	1209.58	6.7	120.24	3154.94	3.8	94.26	3427.47	2.8
**Place of residence**									
Rural	81.94	1091.56	7.5	243.90	1163.10	21.0	157.23	1398.69	11.2
Urban	80.08	2065.54	3.9	107.06	2249.86	4.8	193.31	2611.41	7.4
**Type of health facility**									
Public hospital	76.36	1190.72	6.4	204.64	1173.21	17.4	67.07	1409.77	4.8
Private hospital	102.31	1049.11	9.8	364.25	1523.12	23.9	289.40	1779.87	16.3
**Type of ward**									
Free	69.94	1142.50	6.1	162.79	1185.51	13.7	58.09	1358.91	4.3
Paid	158.68	1281.05	12.4	472.63	1414.69	33.4	326.70	1707.15	19.1
**Duration of stay in hospital**									
Up to 5 days	44.27	1172.83	3.8	61.82	992.92	6.2	93.98	1588.74	5.9
6–10 days	111.34	1159.04	9.6	279.71	1051.67	26.6	179.80	1652.76	10.9
11and above	143.77	1135.87	12.7	342.32	1554.71	22.0	244.00	1048.21	23.3
**Health-care coverage**									
No coverage			–	247.69	1201.14	20.6	165.30	1498.70	11.0
Public			–	203.63	1282.64	15.9	149.40	1474.06	10.1
Private			–			–	22.91	2748.88	0.8
**Total**	81.81	1161.03	**7.0**	234.41	1238.48	**18.9**	159.97	1491.01	**10.7**

Notes: Currency exchange rate of 1 USD=Rupee 82.6321 (Average exchange rate in February 2023: 82.6321) was used for conversion as per values obtained from.

https://www.exchangerates.org.uk/USD-INR-spot-exchange-rates-history2023.html#:~:text=Best%20exchange%20rate%3A%2082.9334%20INR,INR%20on%2022%20Jan%202023.

**Table 3:** Multivariate pooled linear regression results for predicting the effect of selected background characteristics of OOPE for tuberculosis (2004–2018).

Background Characteristics	Coef.	p-value	[95% Conf Interval]		Sig
			Lower	Upper	
**Constant**	8.021	0.00	7.509	8.533	***
**Age (in years)**					
0-14 ®	0				
15–35	−0.322	0.142	−0.753	0.109	
36–59	−0.394	0.056	−0.798	0.011	*
60 and above	0.156	0.624	−0.469	0.781	
**Education**					
Illiterate ®	0				
Up toPrimary	0.137	0.478	−0.242	0.515	
Middle	0.404	0.123	−0.111	0.919	
Secondary and above	−0.082	0.795	−0.707	0.543	
**Gender**					
Male®	0				
Female	0.275	0.092	−0.046	0.596	*
**MPCE quintile**					
Poorest®	0				
Poorer	−0.18	0.455	−0.653	0.294	
Middle	−0.467	0.058	−0.95	0.015	*
Richer	−0.468	0.074	−0.982	0.046	*
Richest	−0.253	0.318	−0.751	0.245	
**Place of residence**					
Rural®	0				
Urban	0.183	0.587	−0.48	0.845	
**Type of health facility**					
Public hospital	0	.	.	.	
Private hospital	0.581	0.049	0.004	1.159	**
**Type of ward**					
Free®	0				
Paid	0.823	0.002	0.303	1.343	***
**Duration of stay in hospital**					
Up to 5 days®	0				
6–10 days	0.837	0.00	0.432	1.242	***
11 and above	1.287	0.00	0.921	1.652	***
**Health-care coverage**					
No	0				
Yes	−0.328	0.071	−0.685	0.029	*

Mean dependent var	8.716	SD dependent var	1.324
R-squared	0.379	Number of obs	202
F-test	6.601	Prob > F	0.000
Akaike crit. (AIC)	625.514	Bayesian crit. (BIC)	685.063

***p < 00.01, **p < 00.05, *p < 00*.1*.

Hardship financing in TB patients above 60 years of age decreased from 37.3% in NSS 60th round (2004) to 17.1% in NSS 75th round (2018) (Table 4). Child TB patients (0–14 years) were most exposed to hardship financing in NSS 60th round (2004) (64.1%) and NSS 75th round (2018) (48.9%). Male patients with TB showed higher exposure to hardship financing compared to females in two rounds [NSS 60th round (2004) and NSS 75th round (2018)]. Shift of households exposed to hardship financing was observed among patients from rural areas in 2004 [37.6% (NSS 60th round), 36.0% (NSS 71st round), and 14.3% (NSS 75th round)] to urban setting was observed in 2018 [34.2% (NSS 60th round), 19.8 (71st round), and 51.0% (75th round)]. Those seeking private health facilities and paid wards for TB treatment showed higher exposure to hardship financing in hospitalisation across all three rounds (Table 4 and S4). The richest Monthly Per-capita Consumer Expenditure (MPCE) quintile had a significant inverse association with hardship financing (OR = 0.087, 95% CI 0.024–0.322, p value = 0.00) (Table 5). Private hospital stay resulted in 4.39 times more hardship financing than in public hospitals (OR = 4.397, 95% CI 1.450–13.336, p value = 0.009). The risk of experiencing hardship financing among patients whose stay was for 11 days or more (OR = 6.948, 95% CI 3.072–15.717, p-value = 0.000) was seven times more than those who stayed up to 5 days. Those having healthcare coverage had slightly higher chances of being exposed to hardship financing (OR = 3.041, 95%CI 0.981–9.424, p-value = 0.054) than those without.

**Table 4:** Percentage of households exposed to hardship financing for TB by selected socioeconomic characteristics in India, (NSS 2004–2018).

Background Characteristics	NSS-2004 (n = 114)	NSS-2014 (n = 106)	NSS-2018(n = 115)
	%	%	%
**Age (in years)**			
0–14	64.1	0.0	48.9
15–35	46.2	41.1	25.7
36–59	33.2	37.2	10.7
60 and above	30.5	31.8	0.2
**Education**			
Illiterate	37.7	12.9	40.4
Up to Primary	36.7	52.3	2.0
Middle	10.4	41.9	25.2
Secondary and above	94.2	44.2	0.3
**Gender**			
Male	39.3	34.0	21.2
Female	33.7	36.2	7.5
**MPCE quintile**			
Poorest	38.5	56.3	20.1
Poorer	68.9	30.4	24.3
Middle	40.3	6.6	29.6
Richer	19.8	7.4	6.7
Richest	9.0	17.2	0.0
**Place of residence**			
Rural	37.6	36.0	14.3
Urban	34.2	19.8	51.0
**Type of health facility**			
Public hospital	28.6	31.9	11.2
Private hospital	70.3	47.6	20.5
**Type of ward**			
Free	32.7	33.6	11.5
Paid	67.5	39.0	26.3
**Duration of stay in hospital**			
Up to 5 days	22.0	9.6	0.0
6–10 days	56.4	41.8	21.6
11and above	57.1	50.5	40.0
**Health-care coverage**			
No coverage	–	36.5	13.0
Public	–	34.0	25.5
Private	–	–	–
**Total**	**37.3**	**34.8**	**17.1**

**Table 5:** Multi-variate logistic regression model for predicting the effect of selected background characteristics of hardship financing among tribal populations with TB in India (2004–2018).

Background Characteristics	Odds ratio	p-value	[95% Conf Interval]	Upper	Significance
			Lower		
**Age (in years)**					
0-14 ®	1				
15–35	1.158	0.806	0.36	3.728	
36–59	0.263	0.061	0.065	1.064	*
60 and above	0.087	0.056	0.007	1.059	*
**Education**					
Illiterate ®	1				
Up to Primary	0.367	0.121	0.103	1.304	
Middle	0.647	0.625	0.113	3.707	
Secondary and above	0.184	0.215	0.013	2.667	
**Gender**					
Male®	1				
Female	0.687	0.493	0.235	2.011	
**MPCE quintile**					
Poorest®	1				
Poorer	0.516	0.323	0.139	1.919	
Middle	0.164	0.011	0.041	0.655	**
Richer	0.203	0.058	0.039	1.058	*
Richest	0.011	0.001	0.001	0.164	***
**Place of residence**					
Rural®	1				
Urban	4.345	0.167	0.542	34.826	
**Type of health facility**					
Public hospital	1				
Private hospital	7.338	0.045	1.05	51.277	**
**Type of ward**					
Free®	1				
Paid	0.573	0.551	0.092	3.572	
**Duration of stay in hospital**					
Up to 5 days®	1				
6–10 days	13.074	0.004	2.304	74.178	***
11 and above	20.603	0.000	4.293	98.87	***
**Health-care coverage**					
No	1				
Yes	3.041	0.054	0.981	9.424	*
**Constant**	0.07	0.004	0.012	0.421	***

Mean dependent var	0.162	SD dependent var	0.369
Pseudo r-squared	0.354	Number of obs	204
Chi-square	63.887	Prob > chi2	0.000
Akaike crit. (AIC)	152.687	Bayesian crit. (BIC)	212.414

***p < 00.01, **p < 00.05, *p < 00*.1*.

15–35 years old in tribal households were most exposed to CHE as per NSS 60th round (2004) and NSS 71st round (2014), whereas the age cohort 0–14 years faced highest CHE in NSS 75th round (2018) (Table 6). Females [NSS 71st round (2014)- 40.5%; NSS 75th round (2018)- 40.8%] compared to males, and MPCE middle quintile households were mostly affected by CHE (NSS 71st round (2014)- 77.9%; NSS 75th round (2018)- 67.1%) were seen to be typically affected by CHE during the latter two rounds. Over 42.2% of hospitalized patients without any healthcare coverage, and 24.5% with public health insurance coverage had exposure to CHE in 2018. Overall, our analysis showed that CHE exposure had increased for tribal households from 2004 to 2014 and then had a downward slide marginally in 2018 (Table 6 and S3). Urban setting had a significant association with exposure to CHE (OR = 0.215, CI = 0.039–1.182, p-value = 0.077). Hospital stays for 6–10 days resulted in 10.21 times more exposure to CHE (OR = 10.214, CI = 1.992–52.362, p-vale = 0.005), in comparison to up to 5 days stay. If the patient had to stay 11 days or more, such a stay resulted in 14.227 times more chances for him to pay CHE (OR = 14.227, CI = 3.153–64.188, p-value = 0.001) than hospital stays of up to 5 days. Those with any healthcare coverage had higher odds to exposure to CHE compared to those without (OR = 1.345, 95% CI 0.404–2.531, p-value = 0.054) (see Table 7).

**Table 6:** Percentage of tribal households exposed to CHE for Tuberculosis by selected socioeconomic characteristics in India, (NSS 2004–2018).

Background Characteristics	NSS-2004 (n = 114)	NSS-2014 (n = 106)	NSS-2018(n = 115)
**Age (in years)**			
0–14	22.4	38.7	53.7
15–35	37.1	49.6	34.9
36–59	12.9	15.5	0.0
60 and above	19.6	0.0	0.0
**Education**			
Illiterate	19.9	19.5	58.9
Up toPrimary	36.6	50.3	17.7
Middle	5.0	46.1	41.1
Secondary and above	94.2	0.3	46.4
**Gender**			
Male	27.5	35.9	36.2
Female	5.5	40.5	40.8
**MPCE quintile**			
Poorest	28.2	54.6	29.9
Poorer	28.0	16.1	26.9
Middle	5.0	77.9	67.1
Richer	43.4	41.9	45.7
Richest	9.6	16.1	10.7
**Place of residence**			
Rural	26.4	38.0	35.1
Urban	0.0	22.0	54.2
**Type of health facility**			
Public hospital	24.5	26.3	13.5
Private hospital	23.7	71.8	80.8
**Type of ward**			
Free	19.3	23.8	11.8
Paid	45.4	64.5	82.9
**Duration of stay in hospital**			
Up to 5 days	9.8	13.8	25.1
6–10 days	29.6	58.7	33.0
11and above	45.9	46.7	62.2
**Health-care coverage**			
No coverage	–	40.1	42.2
Public	–	30.9	24.5
Private	–	–	–
**Total**	**24.3**	**37.3**	**36.9**

**Table 7:** Multi-variate logistic regression model for predicting the effect of selected background characteristics of Catastrophic Health Expenditure among tribal populations with TB in India (2004–2018).

Background Characteristics	Odds ratio	p-value	[95% Conf Interval]		Significance
			*Lower*	Upper	
**Age (in years)**					
0-14 ®	1	.	.	.	
15–35	0.745	0.706	0.162	3.433	
36–59	0.084	0.077	0.005	1.31	*
60 and above	0.381	0.353	0.05	2.922	
**Education**					
Illiterate ®	1	.	.	.	
Up to Primary	1.519	0.579	0.346	6.663	
Middle	1.669	0.579	0.274	10.166	
Secondary and above	5.604	0.117	0.651	48.248	
**Gender**					
Male®	1	.	.	.	
Female	0.907	0.856	0.314	2.616	
**MPCE quintile**					
Poorest®	1	.	.	.	
Poorer	0.462	0.347	0.092	2.309	
Middle	0.522	0.454	0.095	2.862	
Richer	0.61	0.542	0.124	2.993	
Richest	0.537	0.444	0.109	2.64	
**Place of residence**					
Rural®	1	.	.	.	
Urban	0.218	0.072	0.042	1.144	*
**Type of health facility**					
Public hospital	1	.	.	.	
Private hospital	1.307	0.782	0.196	8.725	
**Type of ward**					
Free®	1	.	.	.	
Paid	1.892	0.475	0.329	10.879	
**Duration of stay in hospital**					
Up to 5 days®	1	.	.	.	
6–10 days	9.113	0.006	1.89	43.945	***
11 and above	13.537	0.001	3.113	58.871	***
**Health-care coverage**					
No	1				
Yes	1.345	0.054	0.404	2.531	
Constant	0.158	0.056	0.024	1.051	*

Mean dependent var	0.381	SD dependent var	0.488
Pseudo r-squared	0.237	Number of obs	97
Chi-square	30.501	Prob > chi2	0.016
Akaike crit. (AIC)	132.464	Bayesian crit. (BIC)	176.234

***p < 00.01, **p < 00.05, *p < 00*.1*.

Percentage of the tribal households falling below the PL and the average percentage deficit from the poverty line (PL) due to OOPE for TB shows substantial increase from 2004 to 2018 (Table 8). Tribal households in urban areas showed a higher percentage of poverty headcount ratio in 2014 compared to 2004 and 2018. Over 50.7% of the tribal households in urban areas seeking private hospitals for treatment fell below the PL in NSS-2018. Patients in paid wards had considerably high percentage of the population falling below the PL across all the three rounds. Those with no healthcare coverage had substantial percentage of population falling below the PL along with average percentage deficit from the PL in 2018 compared to those with public healthcare coverage (Table 8 and S5).

**Table 8:** Percentage of tribal households falling below poverty line (poverty headcount ratio) and average percentage deficit from the poverty line (poverty gap ratio) due to OOPE for TB treatment in India (NSS 2004–2018).

Background Characteristics	NSS-2004		NSS-2014		NSS-2018	
	Poverty headcount ratio (%)	Poverty gap ratio (%)	Poverty headcount ratio (%)	Poverty gap ratio (%)	Poverty headcount ratio (%)	Poverty gap ratio (%)
**Age (in years)**						
0–14	0.0	0.0	0.0	0.0	34.8	7.9
15–35	5.0	0.3	8.9	0.3	0.0	0.0
36–59	0.0	0.0	0.0	0.0	9.6	0.1
60 and above	9.5	3.5	0.0	3.5	0.0	0.0
**Education**						
Illiterate	6.3	1.4	21.1	1.4	18.7	3.3
Up toPrimary	0.0	0.0	1.5	0.0	11.1	14.8
Middle	3.3	0.2	12.2	0.2	0.0	0.0
Secondary and above	5.8	0.2	0.3	0.2	44.2	10.6
**Gender**						
Male	5.2	1.0	13.2	1.0	20.2	10.6
Female	4.2	1.5	21.9	1.5	0.0	0.0
**MPCE quintile**						
Poorest	0.0	0.0	0.0	0.0	0.0	0.0
Poorer	0.0	0.0	28.6	0.0	4.8	1.0
Middle	0.0	0.0	25.4	0.0	64.9	51.6
Richer	43.3	9.6	36.3	9.6	24.9	6.0
Richest	0.0	0.0	0.0	0.0	0.0	0.0
**Place of residence**						
Rural	5.5	1.2	15.6	1.2	18.7	9.8
Urban	0.0	0.0	20.5	0.0	0.6	0.0
**Type of health facility**						
Public hospital	3.1	0.6	18.1	0.6	3.7	0.5
Private hospital	9.5	2.1	8.6	2.1	50.7	29.1
**Type of ward**						
Free	1.3	0.2	19.3	0.2	3.8	0.5
Paid	20.8	4.9	8.9	4.9	41.0	24.2
**Duration of stay in hospital**						
Up to 5 days	1.2	0.2	30.4	0.2	21.1	5.1
6–10 days	1.1	0.6	1.7	0.6	0.1	0.0
11and above	15.2	3.1	12.6	3.1	40.9	30.1
**Health-care coverage**						
No coverage	–	–	–	–	25.7	9.3
Public	–	–	–	–	3.3	0.3
Private	–	–			–	–
**Total**	**5.0**	**1.1**	**15.8**	**1.1**	**17.0**	**8.9**

Results from the PSM show that for the unmatched sample estimate, households with health insurance were 11% less likely to experience CHE (at 10% threshold) compared to households that had no insurance coverage in 2014 (Table 9). The calculated ATT values in the treated and control groups were 0.30 and 0.38, respectively, which means that after matching, reduction to CHE (at 10% threshold) was attributed to health insurance coverage by 8% margin. The computed ATU values for the intervention and control groups were 0.41 and 0.46 respectively. This means that the participants who did not have any health insurance, if they were covered by some form of health insurance, the chance of CHE (at 10% threshold) would have reduced by 5%. The 2018 estimates indicate that households with health insurance were 3% less likely to experience CHE (at 10% threshold) compared to households that had no insurance coverage based on computed ATT values. However, the chance of CHE (at 10% threshold) would have increased by 13% between treatment and controls as per computed ATU values (Table 9).

**Table 9:** Impact of health insurance on CHE at 10% threshold using Propensity Score Matching (PSM) among tribal populations with TB (NSS 2014, NSS 2018).

Survey Time	Public insurance Vs. No Insurance	Treated	Controls	Difference	S.E.	T-test
NSS, 2014	Unmatched	0.30	0.41	−0.11	0.10	1.08
	ATT	0.30	0.38	−0.08	0.16	0.52
	ATU	0.41	0.46	0.05		
	ATE			0.00		
NSS, 2018	Unmatched	0.21	0.29	−0.08	0.09	0.91
	ATT	0.21	0.24	−0.03	0.16	0.19
	ATU	0.29	0.16	−0.13		
	ATE			−0.10		

Those households that had health insurance were 7% less likely to be impoverished compared to households that had no health insurance (Table 10) in 2018 according to the unmatched estimate. The calculated ATT values in the treated and control groups were 0.03 and 0.24, respectively, which means that after matching, 21% of the households without any health insurance were impoverished compared to non-insured. The estimates based on a 2014 indicate that 9% of health insurance households pushed on poverty compared to 21% of non-insured households, a difference of 12% which shows that those households that had health insurance were 12% less likely to pushed on poverty compared to households that had no insurance coverage in 2014.

**Table 10:** Impact of health insurance on impoverishment using Propensity Score Matching (PSM) among tribal populations with TB (NSS 2014, NSS 2018).

Survey Time	Public insurance Vs. No Insurance	Treated	Controls	Difference	S.E.	T-test
NSS,2014	Unmatched	0.05	0.13	−0.08	0.06	−1.23
	ATT	0.09	0.21	−0.12	0.09	−1.56
	ATU	0.10	0.08	−0.02	.	.
	ATE			−0.06	.	.

NSS, 2018	Unmatched	0.03	0.11	−0.07	0.05	−1.34
	ATT	0.03	0.24	−0.21	0.12	−1.79
	ATU	0.11	0.07	−0.04	.	.
	ATE			−0.09	.	.

## 4 Discussion

The main findings of the study conducted among hospitalized cases for TB in tribal populations are: (a) There is substantial increase in OOPE and healthcare burden due to TB; (b) Utilisation of private hospitals and paid wards lead to higher OOPE, hardship financing, CHE and impoverishment; (c) Households without healthcare coverage were significantly impacted for CHE and impoverishment compared to those with health insurance.

Over two-thirds of the TB cases are seen in the economically productive age group (15–59 years) with high OOPE, and healthcare burden compared to all other age groups across all age-groups. Within this age-group 15–35 age group shows substantial impact of OOPE, HCB, CHE and poverty impact across all three rounds. These findings are similar to the economic burden due to TB in general population as seen in previous studies [18,19].

Illiterate patients who accessed TB treatment were exposed to higher healthcare burden, catastrophic health expenditure, hardship financing, and impoverishment compared to others due to hospitalisation expenses. According to Census of India 2011 data, the literacy rate among tribal people in India was 59%, which is much lower than the national average (73%) with only some improvement from Census of India 2001 [28]. Previous studies have also highlighted poor health literacy among tribal populations in India [29]. While efforts for improving education access in the country by the government is underway it may be important it might be worthwhile to improve the health literacy in tribal populations for increasing their understanding of need of care for managing TB.

Results from our PSM analysis show that health insurance has a high impact in reducing CHE and impoverishment resulting due to hospitalisation expenditure due to TB. However, almost 61.5% and 66.8% are not covered with any kind of public health insurance scheme as per our analysis. In 2022, the central government has announced the provision of health insurance cover to Renotified, Nomadic and Semi-nomadic trial communities under the Ayushman Bharat Pradhan Mantri Jan Arogya Yojana (AB-PMJAY) under the Scheme for Economic Empowerment of De-Notified, Nomadic and Semi Nomadic Tribes (SEED) [30]. However, analysis done for the Tribal Health in India Report (2018) mentions about substantial under-spending of the Tribal Sub-Plan (TSP) and low earmarked allocation to tribal health [31]. There is a need for faster implementation of this scheme and proper allocation of the TSP to ensure that tribal households receive financial protection to manage TB related expenditure in the future.

Our analysis on CHE using multivariate logistic regression and PSM shows contrasting results.

The Tribal Health in India Report has highlighted the usage of private healthcare facilities among tribal populations in the country [31]. Our analysis shows that the use of private healthcare facilities for TB hospitalisation showed a downward trend from 2004 to 2014 (21%–18.7%) but this has increased to 29.7% in 2018. Our analysis also suggests that the use of private healthcare facilities for TB hospitalisation treatment by tribal populations is substantially lower (29.7%) compared to use of public facilities (70.4%). Additional analysis shows that 31.46% and 28.57% of patients have healthcare coverage to those visiting public and private health facilities respectively. However, this is worrisome, since as per our estimates, patients in private hospitals had substantial OOPE, hardship financing, CHE, and impoverishment. The health outcomes of tribal communities, which enhance the local spread of infectious illnesses like tuberculosis, have been shown to been impacted due to factors related to access to public healthcare services such as distance, and waiting time, in comparison to those living in rural and urban regions [32].

Our multivariate analysis found that populations with health insurance were significantly associated with lower OOPE but had higher odds of exposure to hardship financing and CHE (at 10% threshold). Our estimates points to the methodological aspects to be considered while estimating CHE and impoverishment with limited information about the consumption expenditure by using the capacity-to-pay approach using NSS data [33]. CHE and impoverishment among the poorest and poorer households have remained consistently high over NSS-2004, NSS-2014, and NSS-2018 [33]. Additionally, health insurance has not reduced CHE and impoverishment among poorest, poorer and middle MPCE quintiles. Over 75% of the TB patients across all the three rounds belong to these MPCE categories which could explain the similarity between our results compared to Mohanty & Dwivedi (2021) [33].

The strength of our study lies in the use of nationwide population-based study with multiple time-points, allowing representation and comparison of estimates and thus allowing analysis of trends over the last two decades. We used standardized methods in all these rounds that allow a valid comparison of results.

However, this study has some limitations as well. This study being cross-sectional in nature was not able to capture expenditure for the entire duration of TB treatment which might lead to underestimation of total costs. Self-reporting of illness and costs of care along with responder details could also be potential limitations as observed with any health services utilisation study. The lack of information of clinical profile of TB patients such as drug utilisation, any co-morbidities and TB severity could lead to potential confounding. Our study used hardship financing for measuring the impact of the sale of assets or borrowings on interest, the long-term loss of capital or interest payable was not captured due to paucity of data. We could not use a multidimensional poverty index (MDPI) due to lack of data availability which could underestimate our estimates of CHE and impoverishment among tribal populations with TB. Future studies will need to ensure that these aspects are captured.

## 5 Policy recommendations

**Fig. 1:**
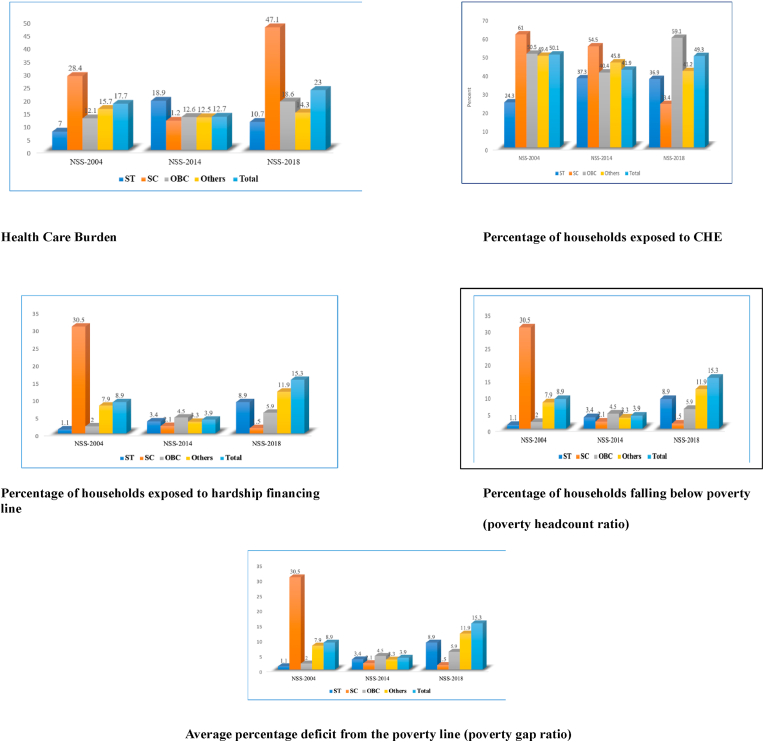
Comparision of healthcare burden, percentage of households exposed to CHE, percentage of households exposed to hardship financing, and percentage of households falling below poverty (poverty headcount ratio) among various social groups due to TB hospitalisation across NSSO rounds (2004–2018).

Our study findings have important policy implications as tribal populations show a substantial percentage of households with TB patients exposed to HCB, CHE, hardship financing, and impoverishment effects compared to other vulnerable social groups [scheduled castes (SC), and other backward castes (OBC)] (Fig. 1, Supplementary Table). This is important to consider as poverty levels are highest among India's tribal population (81.4%), followed by SC (65.8%), and OBC (58.3%) compared to rest of population (33.3%) [34]. With regards to vulnerable populations such as tribal populations, there is increasing need for improving the Direct Benefit Scheme for cash transfer and AB-PMJAY scheme for health insurance coverage in order to offset the OOPE impact of TB among tribal populations in India. Improving the reach of these programmes to tribal areas of the country could be one of ways for reducing the healthcare expenditure impact due to TB. Private hospitals in urban areas need to have better collaboration with government TB units for extending the public TB programs to migrant populations many of whom arrive from tribal areas.

## 6 Conclusion

There is a worsening trend among tribal households in India with members suffering from TB being exposed to significant OOPE due to hospitalisation expenditure resulting in catastrophic health expenditure and impoverishment. The authors recommend strongly for the increasing public expenditure on health, improved adherence to Tribal Sub-Plan (TSP) guidelines, earmarking percentage of Ministry of Tribal Affairs (MOTA) allocation for health and better targeting of direct benefit transfer and health insurance schemes along with a healthy private-public partnership for improving access to TB services for tribal populations in the country.

## Funding

No funding sources.

## Data availability statement

Publicly available dataset was analyzed for this study.

## Ethics statement

The present study used the data from secondary sources which is freely available to individuals. Therefore, no ethical approval is required separately for the present study.

## Declaration of competing interest

The authors declare that they have no conflict of interest.
