# Supplementary material for: Hospitalisation expenditure on tuberculosis among tribal populations in India: A repeated cross-sectional analysis of national sample survey data, 2004 to 2018
Source: Public Health Pract (Oxf). 2024 Mar 5;7:100490. doi: 10.1016/j.puhip.2024.100490 (PMC10958113; doi:10.1016/j.puhip.2024.100490)
Supplement: Multimedia component 1 [file mmc1.doc]

**Supplementary tables**

**Table S1. Profile of tuberculosis-affected individuals (NSS 2004-2018)**

| **Caste** | **Hospitalization** | | | | | |
| --- | --- | --- | --- | --- | --- | --- |
|  | **NSS-2004** | | **NSS-2014** | | **NSS-2018** | |
|  | **%** | **n** | **%** | **n** | **%** | **n** |
| ST | 9.7 | 114 | 10.8 | 106 | 10.3 | 115 |
| SC | 32.3 | 239 | 23.8 | 143 | 25.8 | 123 |
| OBC | 36.5 | 308 | 41.1 | 231 | 39.0 | 220 |
| Others | 21.5 | 206 | 24.4 | 130 | 25.0 | 152 |
| **Total** | **100** | **920** | **100** | **610** | **100** | **683** |

*Source: Author’s computations based on NSS data*

*Note: All ‘n’ are unweighted. Total may not be equal due to some missing cases.*

**Table S 2. Average Expenditure (in USD) and Health Care Burden of Tuberculosis (NSS 2004-2018)**

| **Caste** | **Hospitalization** | | | | | | | | |
| --- | --- | --- | --- | --- | --- | --- | --- | --- | --- |
|  | **NSS-2004** | | | **NSS-2014** | | | **NSS-2018** | | |
|  | **OOPE (in USD)** | **YCE**  **(in USD)** | **Health Care Burden (%)** | **OOPE (in USD)** | **YCE**  **(in USD)** | **Health Care Burden (%)** | **OOPE (in USD)** | **YCE**  **(in USD)** | **Health Care Burden (%)** |
| ST | 81.81 | 1161.03 | 7.0 | 234.41 | 1238.48 | 18.9 | 159.08 | 1482.66 | 10.7 |
| SC | 424.59 | 1494.46 | 28.4 | 170.86 | 1525.95 | 11.2 | 903.63 | 1919.35 | 47.1 |
| OBC | 204.67 | 1695.50 | 12.1 | 286.38 | 2272.56 | 12.6 | 341.21 | 1833.99 | 18.6 |
| Others | 311.00 | 1985.37 | 15.7 | 290.21 | 2315.83 | 12.5 | 293.53 | 2048.10 | 14.3 |
| **Total** | **291.01** | **1640.69** | **17.7** | **254.10** | **1993.45** | **12.7** | **430.11** | **1873.25** | **23.0** |

***Source:*** *Author’s computation based on NSS data.*

*Notes: Health Care Burden = [Average total expenditure (in USD) for hospitalized treatment per hospitalization case during a period of 365 days/Yearly Consumption Expenditure (YCE in (in USD`)] *100.*

**Table S3. Percentage of households exposed to CHE for Tuberculosis (NSS 2004-2018)**

| **Caste** | **Hospitalization** | | |
| --- | --- | --- | --- |
|  | **NSS-2004** | **NSS-2014** | **NSS-2018** |
| ST | 24.3 | 37.3 | 36.9 |
| SC | 61.0 | 54.5 | 23.4 |
| OBC | 50.5 | 40.4 | 59.1 |
| Others | 49.4 | 45.8 | 41.2 |
| **Total** | **50.1** | **41.9** | **49.3** |

*Source: Author’s computation based on NSS data*

**Table S4. Percentage of households exposed to hardship financing for TB treatment care expenditure (NSS 2004-2018)**

| **Caste** | **Hardship Financing** | | |
| --- | --- | --- | --- |
|  | **Hospitalization** | | |
|  | **NSS-2004** | **NSS-2014** | **NSS-2018** |
| ST | 37.9 | 34.8 | 17.1 |
| SC | 70.2 | 36 | 11.3 |
| OBC | 54.3 | 25.4 | 26.3 |
| Others | 44.2 | 16.3 | 19.8 |
| **Total** | **48.7** | **26.7** | **18.13** |

*Source: Authors’ computation based on NSS data*

**Table S5. Percentage of households falling below poverty line (poverty headcount ratio) and average percentage deficit from the poverty line (poverty gap ratio) due to OOPE for TB treatment (NSS 2004-2018)**

| **Caste** | **Hospitalization** | | | | | |
| --- | --- | --- | --- | --- | --- | --- |
|  | **NSS-2004** | | **NSS-2014** | | **NSS-2018** | |
|  | **Poverty headcount**  **ratio (%)** | **Poverty gap ratio (%)** | **Poverty headcount**  **ratio (%)** | **Poverty gap ratio (%)** | **Poverty headcount**  **Ratio (%)** | **Poverty gap ratio (%)** |
| ST | 5.0 | 1.1 | 15.8 | 3.4 | 17.0 | 8.9 |
| SC | 26.2 | 30.5 | 8.6 | 2.1 | 10.9 | 1.5 |
| OBC | 10.1 | 2.0 | 13.7 | 4.5 | 9.6 | 5.9 |
| Others | 20.1 | 7.9 | 8.1 | 3.3 | 23.8 | 11.9 |
| **Total** | **15.0** | **8.9** | **11.0** | **3.9** | **23.2** | **15.3** |

*Source: Author’s computation*
